# Eucalyptol ameliorates Snail1/β-catenin-dependent diabetic disjunction of renal tubular epithelial cells and tubulointerstitial fibrosis

**DOI:** 10.18632/oncotarget.22311

**Published:** 2017-10-16

**Authors:** Dong Yeon Kim, Min-Kyung Kang, Sin-Hye Park, Eun-Jung Lee, Yun-Ho Kim, Hyeongjoo Oh, Yean-Jung Choi, Young-Hee Kang

**Affiliations:** ^1^ Department of Food and Nutrition, Hallym University, Chuncheon, Korea

**Keywords:** β-Catenin, cell-cell disjunction, eucalyptol, Snail, tubulointerstitial fibrosis, Pathology Section

## Abstract

Renal tubulointerstitial fibrosis is an important event in the pathogenesis of diabetic nephropathy. Under pathologic conditions, renal tubular epithelial cells undergo transition characterized by loss of cell-cell adhesion and increased cell migration. This study investigated that eucalyptol inhibited tubular epithelial cell disjunction and tubulointerstitial fibrosis stimulated by glucose. Human renal proximal tubular epithelial cells were incubated for up to 72 h in media containing 27.5 mM mannitol as osmotic controls or 33 mM glucose in the presence of 1-20 μM eucalyptol. Nontoxic eucalyptol inhibited glucose-induced expression of the mesenchymal markers of N-cadherin and α-smooth muscle actin, whereas the induction of E-cadherin was enhanced. Eucalyptol attenuated the induction of connective tissue growth factor and collagen IV by glucose, whereas the membrane type 1-matrix metalloproteinase expression was enhanced with reducing tissue inhibitor of metalloproteinase-2 expression. Oral administration of 10 mg/kg eucalyptol to db/db mice for 8 weeks blunted hyperglycemia and proteinuria. Eucalyptol reversed tissue levels of E-cadherin, N-cadherin and P-cadherin and the collagen fiber deposition in diabetic kidneys. Eucalyptol attenuated the induction of Snail1, β-catenin and integrin-linked kinase 1 (ILK1) in glucose-exposed tubular cells and diabetic kidneys, and the glycogen synthase kinase (GSK)-3β expression was reversely enhanced. Glucose prompted TGF-β1 production in tubular cells, leading to induction of Snail1, β-catenin and ILK1, which was dampened by eucalyptol. Furthermore, the Snail1 gene deletion encumbered the β-catenin induction in glucose/eucalyptol-treated tubular cells accompanying enhanced GSK-3β expression. Therefore, eucalyptol may antagonize hyperglycemia-induced tubular epithelial derangement and tubulointerstitial fibrosis through blocking ILK1-dependent transcriptional interaction of Snail1/β-catenin.

## INTRODUCTION

Diabetic nephropathy (DN), the most common risk factor of end-stage renal disease, incurs glomerular scarring and nephrotic syndrome owing to long-standing hyperglycemia [1, 2]. DN is a multifactorial disorder resulting in glomerulosclerosis including mesangial expansion and glomerular basement membrane thickening [3, 4]. In progressive DN the glomerular filtration barrier is increasingly damaged, leading to persistent proteinuria and abnormal renal function [5, 6]. Some proteins escaped into the urine influence renal tubular cells and instigate interstitial scarring ultimately leading to tubular fibrosis [7]. Tubulointerstitial fibrosis and renal failure develop via activation of epithelial-mesenchymal transition (EMT) process of the tubules in advanced stages of DN [8-10]. Proximal tubular epithelial cells transform into fibroblast-like cells (myofiborblasts) with mesenchymal phenotypes, subsequently migrating into the interstitial spaces and generating extracellular matrix (ECM) proteins such as collagen and fibronectin [11, 12]. The myofibroblast transdifferentiation of proximal tubular epithelial cells is an event underlying diabetes-associated progressive chronic kidney disease [13]. Aberrant EMT-associated fibrosis is considered as a potential mechanism for the chronic renal failure in DN [8, 10, 12]. Thus, tubular epithelial transdifferentiation-related signaling pathways are a target to slow down or prevent unfavorable renal fibrosis.

Current understanding of cellular and molecular mechanisms of renal fibrosis provides multifaceted insights into the development of new therapeutic strategies [14]. Several studies have directly targeted several signaling pathways involving transforming growth factor (TGF)-β, basic fibroblast growth factor (FGF)-2 and connective tissue growth factor (CTGF), and Wnt/β-catenin [14-16]. Although inflammatory cytokines and various growth factors orchestrate the fibrotic process in injured kidney, TGF-β plays a central role in the process [17, 18]. TGF-β is known to induce loss of epithelial cell-cell adhesion markers, acquisition of mesenchymal markers, and increased expression of matrix proteins in epithelial cells [13, 18, 19]. Accordingly, the increased TGF-β in diabetes is an unequivocal candidate in the development of DN that may result in the loss of renal function throughout the nephrons [12, 19]. On the other hand, Snail family members have been shown to induce EMT [20], and TGF-β stimulates synthesis of Snail promoting formation of β-catenin-T-cell factor-4/lymphoid enhancer factor-1 transcription complexes [21]. The signaling mechanism entailing the convergence of TGF-β and β-catenin signaling confers loss of cell–cell adhesion and acquisition of the mesenchymal phenotype [19, 21, 22]. One can assume that the collaborative signaling of Snail and β-catenin is involved in fostering renal fibrosis associated with diabetes.

Numerous studies have shown inhibitory effects of natural compounds on renal dysfunction in DN [23, 24]. Natural compounds that manipulate epithelial transdifferentiation can be exploited in developing therapies targeting against renal tubulointerstitial fibrosis [25, 26]. Several compounds ameliorate fibrosis through encumbering TGF-β/Smad signaling pathway. Epigallocatechin-3-gallate and α-lipoic acid inhibit renal interstitial fibrosis in a mouse model of unilateral ureteral obstruction by alleviation of inflammatory responses and blockade of TGF-β-Smad activation [27, 28]. In contrast, curcumin attenuates TGF-β1-induced transdifferentiation of renal tubular epithelial cells via ERK- and peroxisome proliferator-activated receptor γ-dependent pathway but not Smad-dependent Pathway [25]. In addition, ferulic acid inhibits TGF-β1-induced fibrosis in renal proximal tubular epithelial cells by inhibiting Smad-integrin-linked kinase (ILK1)-Snail pathway [29]. Quercetin blocks high glucose-induced EMT of renal tubular proximal epithelial cells and diabetic renal fibrosis, which entails the inhibition of Snail and the activation of mTORC1/p70S6K signaling [30]. However, potential mechanisms underlying antagonistic effects of natural compounds on diabetes-associated renal tubulointerstitial fibrosis remain largely unknown.

**Figure 1:**
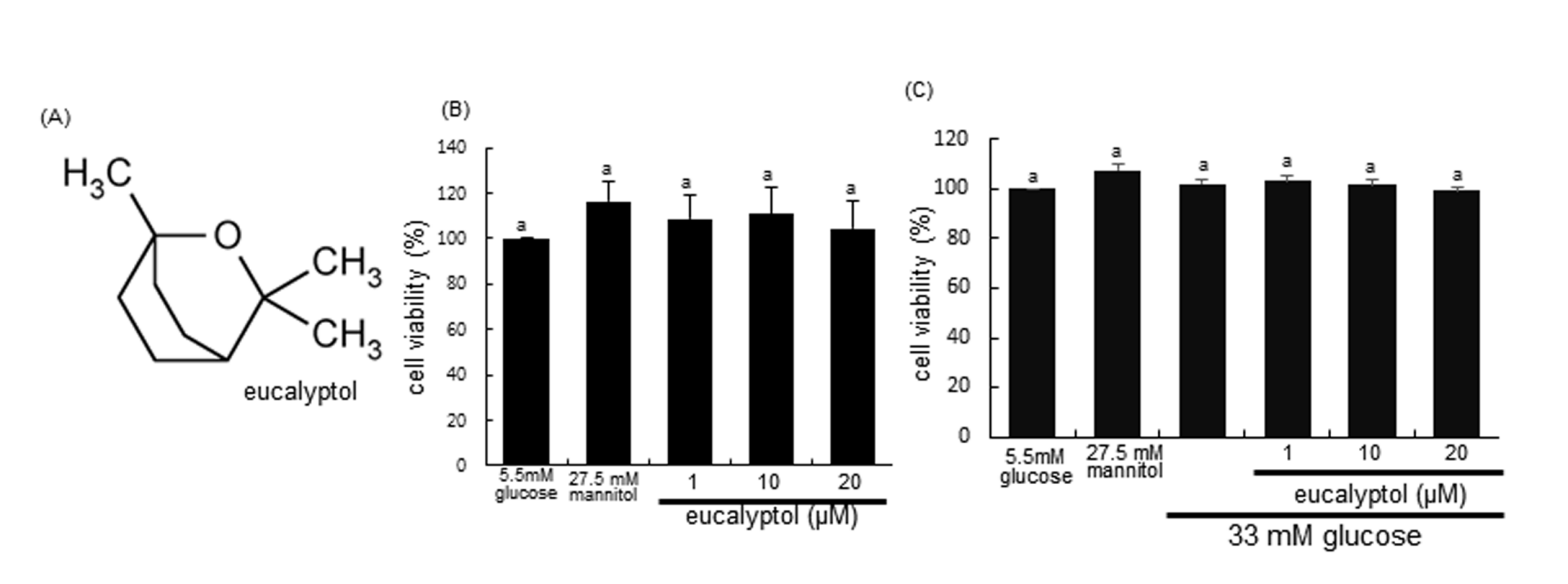
Chemical Structure of eucalyptol (**A**), cytotoxicity of 1-20 μM eucalyptol (**B**), and cell viability of eucalyptol under high glucose milieu (**C**). Human renal proximal tubular epithelial cells (RPTEC) were cultured for 72 h with high glucose (33 mM glucose) in the absence and presence of 1-20 μM eucalyptol. Cells were incubated with 5.5 mM glucose plus 27.5 mM mannitol as an osmotic control. The RPTEC viability was measured by MTT assay. Bar graphs for viability (mean ± SEM, n=4) was expressed as percent cell survival compared to untreated cells. Values in bar graphs not sharing a letter are different at P<0.05.

Eucalyptol (1,8-cineole, Figure 1A) is a naturally occurring organic essential oil that is a cyclic ether and a monoterpenoid chiefly present in eucalyptus oil. Also, it is found in bay leaves, tea tree, rosemary, and other aromatic plant foliage. Eucalyptol has been well-documented to be involved in various pharmacological activities including inflammation and pain relief in numerous products of mouthwash and cough suppressant [31-33]. This compound suppresses human colorectal cancer proliferation by inducing apoptosis in human colon cancer cells [34]. Additionally, essential oil extracts with eucalyptol from black pepper inhibits activities of α-amylase, α-glucosidase and angiotensin-1 converting enzyme relevant to type 2 diabetes and hypertension [35]. However, it is still elusive whether eucalyptol improves renal structure and function by inhibiting renal fibrosis in DN. The present study investigated that eucalyptol improved cell-cell adhesion and mitigated diabetic fibrosis in high glucose-exposed renal proximal tubular epithelial cells (RPTEC) and in db/db mice. E-cadherin loss and α-SMA induction, and collagen fiber accumulation were elucidated for the antagonistic effects of eucalyptol on diabetic fibrosis. Furthermore, this study examined the involvement of TGF-β1-dependent transcriptional signaling of Snail1 and β-catenin in diabetes-associated fibrosis. Eucalyptol merits further exploration as a therapeutic agent in the prevention and treatment of tubular cell-cell dysfunction- and tubulointerstitial fibrosis-linked chronic kidney diseases.

## RESULT

### Inhibition of glucose-induced loss of epithelial cell-cell adhesion by eucalyptol

Loss of renal tubular epithelial cell-cell adhesion and epithelial transdifferentiation are events underlying progressive chronic kidney disease in diabetes [13]. E-cadherin is a cell-cell adhesion molecule crucial for epithelial behavior and an invasion suppressor down-regulating motility of the cells [36]. The current study investigated that eucalyptol blocked loss of epithelial cell-cell adhesion due to transdifferentiation of epithelial cells to myofibroblasts. RPTEC exposed to high glucose for 72 h increased the N-cadherin induction, while epithelial expression of E-cadherin was diminished (Figure 4A). However, the addition of 1-20 μM eucalyptol attenuated the N-cadherin induction in a dose-dependent manner and reversed the E-cadherin expression dampened by high glucose (Figure 4A). Furthermore, the Cy3-immunocytocheminal red-staining confirmed that eucalyptol highly enhanced the expression of E-cadherin in cells exposed to high glucose (Figure 4B). On the other hand, ≥10 μM eucalyptol dampened the expression of α-SMA, a marker of myofibroblast formation, elevated following the 72 h-stimulation of tubular cells by high glucose (Figure 4A), indicating that eucalyptol inhibited the transdifferentiation of renal tubular epithelial cells. One can assume that eucalyptol may encumber glucose-induced transition morphology to fibroblastic phenotype in epithelial cells.

The *in vivo* data supported the *in vitro* results that eucalyptol inhibited high glucose-induced renal tubular epithelial transdifferentiation. Oral administration of 10 mg/kg eucalyptol restored the E-cadherin induction demoted in diabetic renal tissues, and reduced the tissue levels of N-cadherin (Figure 4C). Additionally, the treatment of eucalyptol blocked the α-SMA induction in diabetic kidneys (Figure 4C). Thus, eucalyptol may improve hyperglycemia-associated renal tubular epithelial barrier dysfunction.

### Blockade of renal tubular accumulation of matrix proteins by eucalyptol

Tubulointerstitial fibrosis represents an abnormal accumulation of connective collagen fibres in renal tubular tissues, and is known to promote adult-onset diabetic kidney diseases [37]. This study attempted to determine whether eucalyptol alleviated diabetes-associated tubulointerstitial fibrosis. The induction and production of fibrosis-related CTGF and collagen IV by high glucose were dose-dependently allayed by adding eucalyptol to RPTEC (Figure 5A). The expression and localization of MMP enzymes in the kidney are linked to various renal diseases such as acute kidney injury, glomerulosclerosis, tubulointerstitial fibrosis, chronic allograft nephropathy and renal cell carcinoma [38]. Also, this study evaluated that eucalyptol inhibited hyperglycemia-prompted deposition of matrix proteins. High glucose diminished the epithelial expression of matrix-degrading MT1-MMP, while the cellular induction of its inhibitor TIMP-2 was elevated in tubular epithelial cells (Figure 5B). It was found that eucalyptol dose-dependently commenced the MT1-MMP expression halted by high glucose. On the contrary, this compound demoted the TIMP-2 expression increased in tubular epithelial cells exposed to 33 mM glucose (Figure 5B).

This study further investigated that eucalyptol inhibited the structural remodeling of renal tubules caused by increased deposition of matrix proteins. Masson’s trichrome staining revealed that dense collagen fiber deposition was notably observed (blue color) in tubulointerstitial tissues of db/db mouse kidneys (Figure 5C). When 10 mg/kg eucalyptol was orally supplemented to db/db mice, the collagen fiber deposition was suppressed, and was comparable to, if not indistinguishable from that of the db/m control mice (Figure 5C). Thus, eucalyptol may alleviate tubular subepithelial fibrosis, indicating a novel therapeutic agent antagonizing diabetic fibrotic diseases.

### Modulation of tubular fibrosis-associated Snail1 signaling by eucalyptol

Snail1, a member of the Snail superfamily of zinc-finger transcription factors, is expressed during renal embryogenesis and plays pivotal roles in renal fibrogenesis via suppressing E-cadherin [39]. The Snail1 induction in cortical tubules of diabetic kidneys was evaluated by light microscopy using a specific antibody of Snail1. There was heavy staining in renal tubules of db/db mice observed, indicating that the Snail1 expression was highly elevated in diabetic kidney tubules (Figure 6A). In contrast, the Snail1 expression was diminished in eucalyptol-treated db/db mice, which was indistinguishable from that of db/m controls. In db/db mouse kidneys with highly-reduced P-cadherin, the renal tissue levels of ILK1 and β-catenin markedly increased, but the GSK-3β level decreased (Figure 6B), indicating that the tubular fibrosis was mediated via ILK1/β-catenin signaling in diabetic kidney. However, the eucalyptol treatment antagonized renal tubular cell-cell dysfunction through attenuating expression of ILK1 and β-catenin with increasing the tissue level of GSK-3β (Figure 6B).

High glucose significantly induced the expression of Snail1 and β-catenin in RPTEC, which was attenuated by treating eucalyptol to these cells (Figure 7A). The FITC-immunofluorescent green staining revealed that the nuclear translocation of Snail1 was enhanced in tubular cells due to high glucose (Figure 7B). However, such Snail1 translocation was blocked by adding submicromolar eucalyptol to cells. In addition, eucalyptol inhibited the glucose-induced ILK1 expression through enhancing the GSK-3β induction in an inverse way (Figure 7C). Accordingly, eucalyptol may block the ILK1/β-catenin signaling possibly involved in renal tubular fibrosis.

### Blockade tubular fibrosis-linked TGF-β1 signaling by eucalyptol

This study examined that TGF-β1 signaling was linked to ILK1-β-catenin pathway responsible for diabetes-associated renal tubulointerstitial fibrosis, which was disturbed by treating submicromolar eucalyptol to tubular epithelial cells. As expected, the release and cellular expression of TGF-β1 highly increased in 33 mM glucose-exposed RPTEC (Figure 8A and 8B). In contrast, such TGF-β1 production was reversed by treating ≥10 μM eucalyptol to high glucose-experiencing tubular cells. On the other hand, TGF-β1 remarkably elevated the Snail1 induction in RPTEC much higher than 33 mM glucose, which was dampened by 20 μM eucalyptol (Figure 8C). Similar to 33 mM glucose, TGF-β1 increased the expression of ILK1 and β-catenin in tubular cells and 20 μM eucalyptol inhibited the induction of these proteins (Figure 8C). In the presence of 20 ng/ml TGF-β1 kinase receptor the inhibition of E-cadherin expression by glucose was attenuated, and the glucose induction of N-cadherin was diminished (Figure 8D). Therefore, eucalyptol may suspend the TGF-β1-ILK1-β-catenin signaling leading to the Snail1 transactivation in diabetic renal tubules.

### Interaction of Snail1 and β-catenin in diabetic cell-cell dysfunction and tubular fibrosis

This study investigated the transcriptional collaboration of Snail1 and β-catenin in loss of diabetic tubular cell-cell adhesion. This study showed that high glucose/hyperglycemia enhanced the renal tubular induction of Snail1 and β-catenin (Figure 6A/6B and Figure 7A). Consistently, high glucose diminished the E-cadherin induction with increasing Snail1 expression, which was significantly reversed by 20 μM eucalyptol (Figure 9A). When the Snail1 gene was deleted in RPTEC transfected with Snail1 siRNA, the E-cadherin expression was not reduced by the presence of high glucose. Also, 20 μM eucalyptol did not influence the E-cadherin expression (Figure 9A). Accordingly, glucose-induced loss of cell-cell adhesion may entail the Snail1 induction in renal tubular cells.

Snail and β-catenin proteins are known to be cooperatively involved in transcriptional episode that control fibrosis in tandem fashion [40]. The β-catenin induction by high glucose was attenuated in Snail1 gene-absent RPTEC, which was abolished by 20 μM eucalyptol (Figure 9B). It was assumed that Snail1 was involved in the β-catenin induction in high glucose-exposed tubular cells. It should be noted that the Snail1 gene deletion did not interfere with the eucalyptol blockade of β-catenin induction (Figure 9B). When the β-catenin gene was silenced in RPTEC, the Snail1 induction by high glucose was attenuated (Figure 9C). Also, the β-catenin gene deletion did not affect the Snail1 inhibition and the E-cadherin induction by eucalyptol. One investigation shows that ILK1 can control β-catenin-dependent expression of Snail in APC7/7 human colon carcinoma cells by distinct pathways [41]. The glucose induction of ILK1 was not disturbed even in Snail1 gene-deleted tubular cells (Figure 9B). It has been shown that GSK-3β regulates Snail and β-catenin expression during Fas-induced EMT [42]. This study showed that the GSK-3β induction by eucalyptol was further enhanced in Snail1 gene-absent RPTEC (Figure 9B).

## DISCUSSION

Ten major findings were extracted from this study. 1) Oral administration of 10 mg/kg eucalyptol to db/db mice for 8 weeks ameliorated hyperglycemia and proteinuria by reducing plasma glycated hemoglobin level and urinary albumin/creatinine ratio. 2) The addition of 1-20 μM eucalyptol to renal tubular cells dose-dependently antagonized the glucose induction of N-cadherin and α-SMA, while the E-cadherin expression dampened by glucose was restored. 3) Oral administration of 10 mg/kg eucalyptol to db/db mice enhanced the E-cadherin induction but reduced the tissue levels of N-cadherin and α-SMA in diabetic kidneys. 4) The treatment of eucalyptol inhibited renal tubular CTGF induction and collagen IV secretion by glucose, and reversed tubular MT1-MMP expression suppressed by glucose. 5) The tubulointerstitial deposition of collagen fibers was attenuated by supplementing 10 mg/kg eucalyptol to db/db mice. 6) Renal tubular induction of Snail1 and β-catenin was diminished in eucalyptol-treated tubular epithelial cells and diabetic kidneys. 7) Eucalyptol encumbered the Snail1 transactivation in glucose-exposed tubular cells, and attenuated TGF-β1 production leading to marked inhibition of Snail1, ILK1 and β-catenin induction via blockade of TGF-β1 kinase receptors. 8) This compound boosted the ILK1 repression and the GSK-3β induction in tubular epithelial cells and diabetic kidneys. 9) The Snail1 gene deletion abolished the glucose induction of β-catenin in eucalyptol-treated tubular cells congruently with the enhanced GSK-3β expression. 10) The β-catenin gene silencing diminished the Snail1 induction by glucose without inhibiting E-cadherin expression in eucalyptol-treated tubular cells. Accordingly, eucalyptol suppressed hyperglycemia-induced loss of renal tubular cell-cell adhesion and renal tubulointerstitial fibrosis through blocking the interactive induction of Snail1 and β-catenin.

Hyperglycemia-induced inflammatory response plays a crucial role in the occurrence and development of diabetic complications [43]. With inflammatory cytokines and signaling pathways as important mediators, targeting inflammation may be a new avenue for alleviating diabetic complications [44]. Persistent inflammation in renal tissues is emerging as an important mechanism for an important pathophysiological basis for diabetic kidney diseases [17, 18, 45]. In addition, hyperglycemia induces expression of fibrogenic mediators resulting in renal fibrosis, which leads to loss of renal function and proteinuria [6, 14, 16]. Tubular induction of chemokines and cytokines and complement activation evoke inflammatory cell infiltration and fibrogenesis in the tubular interstitium [7]. Excess glucose and proteins in tubules may directly contribute to chronic tubulointerstitial fibrosis, one of the key factors that induce the renal damage, favoring the self-destruction of the kidney structure [37, 46]. Key extracellular conditions that contribute to damage to the proximal tubule exert their effects through changes in TGF-β signaling, renin-angiotensin axis, formation of advanced glycation end products, and dysregulation of multiple pathways such as polyol pathway, hexosamine pathway and protein kinase C pathway [47]. As expected, this study revealed that there were the collagen fiber deposition and fibrosis in renal tubular tissues and albuminuria apparent in diabetic animals. The possible mechanisms of renal proximal tubular fibrosis can facilitate the development of specific therapeutic targets of the kidney.

The EMT is a process by which epithelial cells lose their cell polarity and cell-cell adhesion, and acquire migratory and invasive properties to form spindle-shaped, elongated mesenchymal cells [19, 48]. This process occurs in organ fibrosis, wound healing, and in the initiation of metastasis for cancer progression [9, 19, 48]. Extensive body of literature suggests that EMT is considered as one source of matrix-generating fibroblasts in the diseased kidney and myofibroblasts play a crucial role in the production of ECM [10, 11]. This study showed the decreased expression of E-cadherin and P-cadherin, and the acquisition of mesenchymal N-cadherin in high glucose-exposed tubular cells and diabetic kidneys. It was assumed that hyperglycemia resulted in the loss of tubular epithelial cell-cell adhesion. Glucose induced α-SMA and ECM proteins of collagen IV and CTGF in tubular epithelial cells, indicating that tubular epithelial cells were transformed into myofibroblasts. Also, the collagen fibers were highly accumulated in the renal tubular interstitium, a typical hallmark of DN. On the other hand, eucalyptol inhibited distinct biochemical alterations in tubular epithelial cell-cell adhesion typical in the EMT process. This compound could suppress the expansion and deposition of matrix-producing cells in diabetic kidneys through blocking phenotypic conversion of tubular epithelial cells.

Potential mechanisms of DN can identify new therapeutic targets with tubular epithelial cell lines that resemble the cell type *in vivo* will [15, 47]. However, true renoprotective mechanisms for the therapeutic targets against tubulointerstitial fibrosis and tubular atrophy remain to be elucidated. Many studies have shown that natural compounds manipulating EMT may be exploited in developing therapies combating renal tubulointerstitial fibrosis and injury in DN [13, 25, 26]. Several naturally-occurring compounds such as chrysin and resveratrol, mitigate renal tubular fibrosis through blocking TGF-β/Smad signaling pathway [24, 26]. Similarly, in a mouse model of unilateral ureteral obstruction the tea polyphenol epigallocatechin-3-gallate suppresses renal interstitial fibrosis through blocking the activation of TGF-β-Smad signaling [27]. In contrast, curcumin, a substance in turmeric, attenuates TGF-β1-induced renal tubular EMT via Smad-independent pathway [25]. Resveratrol blunts renal injury and fibrosis by inhibiting TGF-β pathway on MMP-7 [24]. This study revealed that eucalyptol inhibited glucose-triggered TGF-β1 signaling that might prompt renal tubular epithelial cell disjunction and tubulointerstitial fibrosis. Eucalyptol promoted the MT-1 MMT induction disrupting renal fibrosis by inhibiting TGF-β pathway. Other novel potential therapeutic targets for the treatment of kidney fibrosis include diverse signaling pathways involving fibroblast growth factor-2, CTGF, angiotensin II and Wnt/β-catenin [14, 15]. Eucalyptol abrogated renal tubular epithelial induction of CTGF on collagen IV by glucose.

It has been demonstrated that ILK1 is a critical mediator for tubular EMT and renal interstitial fibrogenesis stimulated by TGF-β1-dependent Smad signaling [49]. Pharmacologic inhibition of ILK signaling attenuates renal interstitial fibrosis through encumbering TGF-β1-induced phosphorylation of Akt and GSK-3β, and cyclin D1 expression [49]. Similarly, eucalyptol inhibited the TGF-β1-dependent ILK1 induction in tubular cells experiencing EMT and renal fibrosis. Quercetin blocks diabetic renal fibrosis induced through the inhibition of Snail and the activation of mTORC1/p70S6K signaling [30]. Ferulic acid suppresses TGF-β1-induced fibrosis by inhibiting Smad-ILK1-Snail pathway [29]. ILK1 can regulate β-catenin-dependent expression of Snail in human colon carcinoma cells, and GSK-3β manipulates the expression of Snail and β-catenin during Fas-induced EMT [41, 42]. Accordingly, the induction of Snail1 and β-catenin might be dependent on the signaling of ILK1 and GSK-3β in glucose-exposed tubular epithelial cells and db/db mouse kidneys. Eucalyptol inhibited glucose-induced tubular epithelial cell derangement through disturbing tandem induction of ILK1- and GSK-3β-dependent Snail1 and β-catenin. Since the Snail1 gene deletion blunted the induction of β-catenin via enhanced induction of GSK-3β but not influencing ILK1 expression. Thus, one can assume that the convergence of Snail1 and β-catenin signaling confers loss of cell–cell adhesion, acquisition of the mesenchymal phenotype and matrix-producing cell transdifferentiation.

In summary, this study investigated the competence of eucalyptol in opposing diabetic kidney diseases characteristic of renal tubular epithelial derangement and tubulointerstitial fibrosis. Nontoxic eucalyptol blocked glucose-prompted loss of E-cadherin, expression of N-cadherin and α-SMA, and induction of CTGF and collagen IV. Eucalyptol attenuated matrix-degrading MT-1 MMP induction in glucose-exposed epithelial cells. Oral supplementation of eucalyptol counteracted myofibroblast-like cell formation and collagen fiber accumulation via the inhibition of α-SMA induction in mouse kidneys. Furthermore, this compound blocked the cooperative signaling of Snail1 and β-catenin that was dependent on the induction of ILK1 and GSK-3β by TGF-β1. Accordingly, the Snail1-β-catenin signaling might be involved in fostering tubular barrier dysfunction and renal fibrosis associated with diabetes. Therefore, eucalyptol was a potent inhibitor of Snail1 and β-catenin combating renal tubulointerstitial fibrosis and tubular epithelial derangement in diabetic models of renal tubular cells and kidneys.

## MATERIALS AND METHODS

### Materials

Renal epithelial cell growth medium, human epidermal growth factor (EGF), hydrocortisone, insulin, transferrin, epinephrine, triiodothyronine, fetal bovine serum (FBS) and GA-1000 were purchased from Lonza (Walkersvillle, MD). Dulbecco’s modified Eagle’s medium (DMEM), nutrient mixture F-12 Ham medium, mannitol, D-glucose and eucalyptol were obtained from Sigma-Aldrich Chemical (St. Louis, MO) as were all other reagents, unless specifically stated elsewhere. Rabbit polyclonal N-cadherin antibody was supplied by Abcam Biochemicals (Cambridge, UK). Rabbit polyclonal antibodies of α-smooth muscle actin (α-SMA), collagen type IV, membrane type 1-matrix metalloproteinase (MT1-MMP) tissue inhibitor of metalloproteinases-2 (TIMP-2), and β-catenin were obtained from Santa Cruz Biotechnology (Santa Cruz, CA). Rabbit polyclonal connective tissue growth factor (CTGF) antibody was provided by Peprotech (Rocky Hill, NJ). Rabbit polyclonal antibodies of E-cadherin, Snail1, integrin linked kinase 1 (ILK1) and TGF-β1, and mouse polyclonal glycogen synthase kinase-3β (GSK-3β) antibody were purchased from Cell signaling Technology (Beverly, CA). Mouse monoclonal β-actin antibody was obtained from Sigma-Aldrich Chemical. Horseradish peroxidase (HRP)-conjugated goat anti-rabbit IgG, goat anti-mouse and donkey anti-goat IgG were purchased from Jackson ImmumnoReserch Laboratories (West Grove, PA). TGF-β1 protein was provided by R&D system (Minneapolis, MN). TGF- β receptor1 kinase inhibitor was obtained from Calbiochem (Darmstadt, Germany).

Eucalyptol (Sigma-Aldrich Chemical) was dissolved in dimethyl sulfoxide (DMSO) for live culture with cells; a final culture concentration of DMSO was <0.5%.

### *In vitro* culture of renal tubular epithelial cells

Human RPTEC (Lonza, Walkersvillle, MD) were cultured at 37°C humidified atmosphere of 5% CO_2_ in air. Routine culture of RPTEC was performed in DMEM plus F-12 (1:1) media containing 0.5% FBS, 2 mM glutamine, 100 U/ml penicillin, 100 μg/ml streptomycin supplemented with 5 μg/ml insulin, 5 μg/ml hydrocortisone, 0.5 μg/ml epinephrine, 10 μg/ml transferrin, 3 ng/ml human EGF and 6.5 ng/ml triiodothyronine. RPTEC in passages of 6-10 were sub-cultured at 90% confluence and used for further experiments with 1-20 μM eucalyptol eucalyptol. RPTEC were incubated for 72 h in media of 5.5 mM glucose, 5.5 mM glucose plus 27.5 mM mannitol, or 33 mM glucose containing 1-20 μM eucalyptol.

After RPTEC were exposed to 33 mM glucose and 1-20 μM eucalyptol, 3-(4,5-Dimetylthiazol-yl)-diphenyl tetrazolium bromide (MTT, Duchefa Biochemie, Haarlem, Netherlands) assay was carried out to quantitate cellular viability. Cell viability of RPTEC was determined using a colorimetric assay based on the uptake of MTT by viable cells. Eucalyptol per se did not show RPTEC toxicity (Figure 1B). When RPTEC were treated with 33 mM glucose for 72 h in the absence and presence of 1-20 μM eucalyptol, there was no alteration in cell viability observed from the MTT assay (Figure 1C).

### *In vivo* animal experiments

Adult male db/db mice (C57BLKS/+Lepr^db^ Iar; Jackson Laboratory, CA) and their age-matched non-diabetic db/m littermates (C57BLKS/J; Jackson Laboratory) were introduced in the current study. To measure food and water intakes, mice were housed conventionally in individual stainless steel hanging wire-mesh cages, with food and tap water provided ad libitum. Mice were kept on a 12 h light/12 h dark cycle at 23 ± 1°C with 50 ± 10% relative humidity under specific pathogen-free conditions, fed a standard pellet laboratory chow diet (Cargill Agri Purina, Biopia, Korea) and were provided at the animal facility of Hallym University. This study included db/db mice at 6 weeks of age because they begin to develop diabetes (hyperglycemia) at the age of 7-8 weeks. The animals were allowed to acclimatize for a week before beginning the experiments. Mice were divided into three subgroups (n = 9 for each subgroup). The first group of mice was non-diabetic db/m control mice, and db/db mice were divided into two groups. One group of db/db mice was orally administrated 10 mg/kg BW eucalyptol daily for 8 weeks. All experiments were approved by the Committee on Animal Experimentation of Hallym University and performed in compliance with the University’s Guidelines for the Care and Use of Laboratory Animals (hallymR1 2016-10). No mice were dead and no apparent signs of exhaustion were observed during the experimental period.

The wet weights of liver, kidney, pancreas and heart of db/db mice were much higher than those of db/m control, while the spleen of diabetic mice was significantly lighter (Table 1). The weights of kidney and heart were reduced by supplementing 10 mg/kg eucalyptol.

**Table 1:** Alterations of organ weights in eucalyptol-treated db/db mice

groups	liver	kidney	pancrease	spleen	heart
db/m	1.123±0.104	0.380±0.012	0.102±0.012	0.042±0.003	0.135±0.009
db/db	1.627±0.093	0.399±0.013	0.214±0.025	0.031±0.003	0.136±0.003
db/db+eucalyptol	1.638±0.076	0.417±0.029	0.198±0.021	0.037±0.004	0.134±0.002

The db/db mice were orally supplemented with 10 mg/kg eucalyptol for 8 weeks. The values in graphs represent as mean ± SEM (n = 9 mice). Values not sharing a common letter are significantly different at P<0.05.

### Sampling of blood and urine

**Figure 2:**
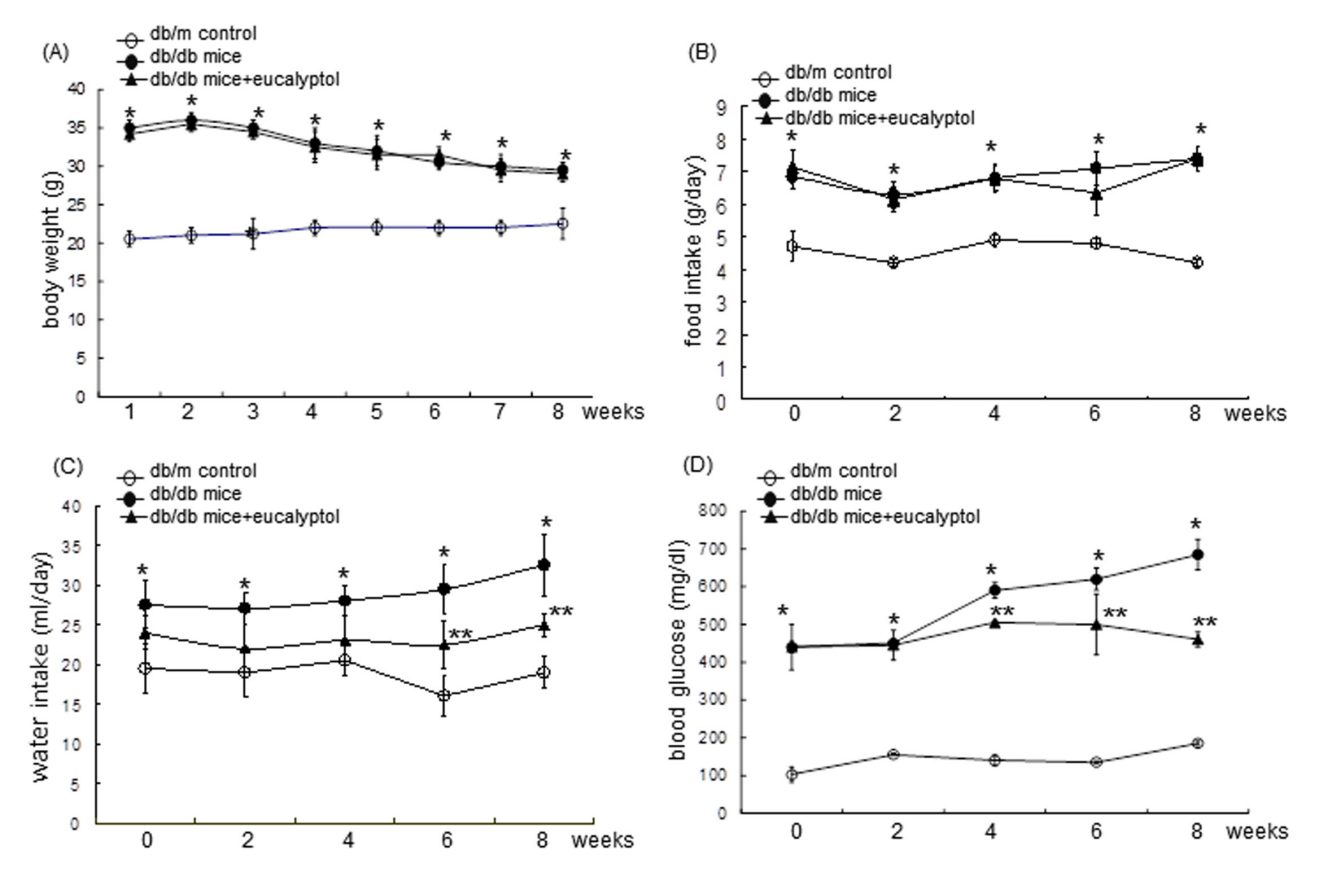
Body weight (**A**), food intake (**B**), drinking water intake (**C**), and fasting blood glucose (**D**) during eucalyptol supplementation. The db/db mice were orally supplemented with 10 mg/kg eucalyptol for 8 weeks. During the oral administration of eucalyptol, the food and water intakes were measured at every third day and the blood glucose was recorded once per week. The values in graphs represent as mean ± SEM (n = 9 mice). *P<0.05 relative to db/m control mice; **P<0.05 relative to db/m control mice and db/db mice.

Body weight, food intake, and drinking water intake were measured in mice every week during the 8 week-eucalyptol supplementation. Food and water intakes were measured daily for 10 weeks. Pre-weighed food was provided in a standard stainless steel hopper. The amount of food remaining including any on the bottom of the cages or any spilled on plastic sheets placed under each cage was measured. Water intake was manually measured by weighing residual amounts in a water bottle. Body weight and food intake of db/db mice were much higher than those of db/m controls regardless of the supplementation of 10 mg/kg eucalyptol (Figure 2A and 2B). However, 10 mg/kg eucalyptol tended to reduce water drinking from the 6^th^ week after its supplementation (Figure 2C). Fasting blood glucose in the mouse tail veins was measured every other week by using a blood glucose meter (ACCU-CHEK Performa, Roche diagnostics, Mannheim, Germany). Plasma glucose levels highly elevated in db/db mice (>4-fold) declined owing to eucalyptol administrated for ≥4 weeks (Figure 2D).

**Figure 3:**
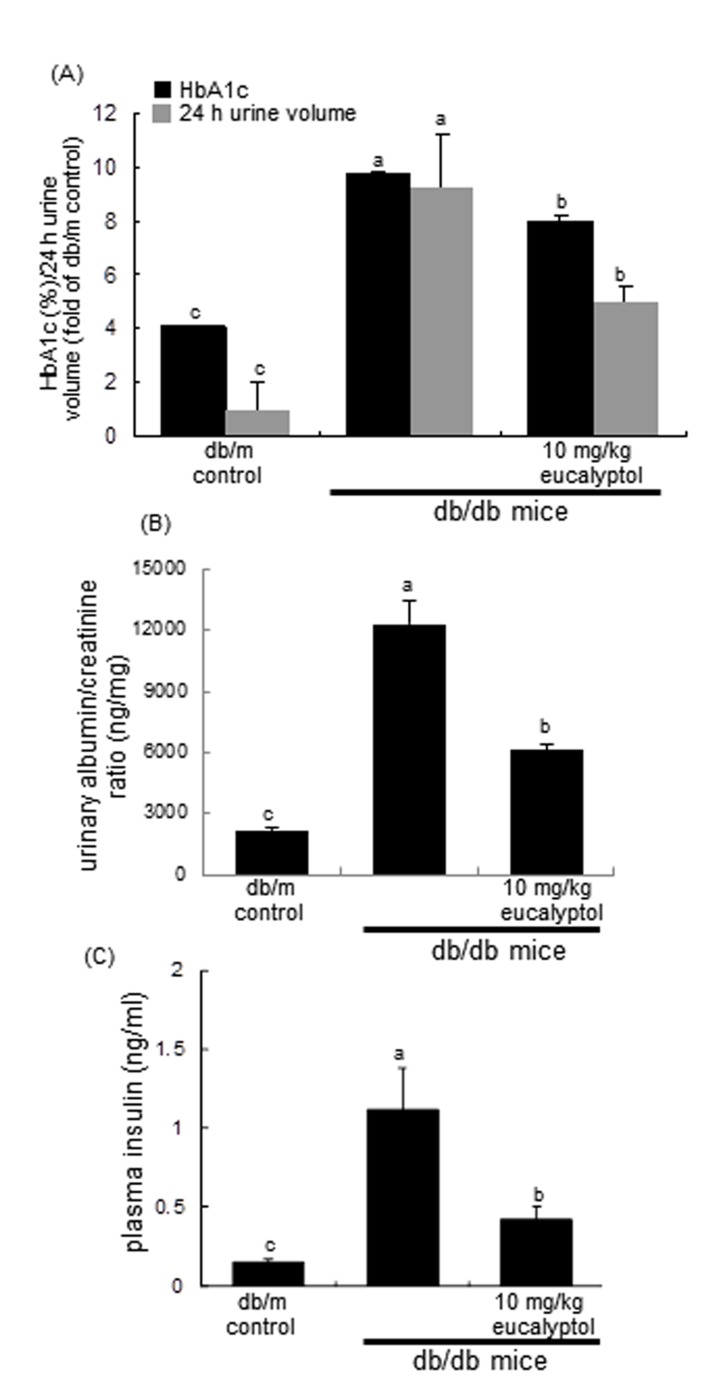
Effects of eucalyptol on glycated hemoglobin level (HbA1c) and urine volume (**A**), urinary albumin/creatinine ratio (**B**), and plasma insulin levels **(C).** The db/db mice were orally supplemented with 10 mg/kg eucalyptol for 8 weeks. Mouse urine volume was collected overnight using metabolic cages. Blood glycated hemoglobin HbA1C was measured by using high-performance liquid chromatography technique (A), and plasma insulin levels were measured by using with ELISA (C). Values (mean ± SEM) in bar graphs not sharing a common letter are significantly different at P<0.05.

The 24 h-urine collection was carried out by using metabolic cages. The 24 h urine volume of db/db mice were much higher (20-fold) than that of db/m controls, while in eucalyptol-administrated animals the volume declined by ≈50% (Figure 3A). Blood glycated hemoglobin HbA1C, a biomarker of development of diabetic complications, was measured by using high-performance liquid chromatography technique. Eucalyptol treatment lowered plasma HbA1C levels markedly elevated in db/db mice (Figure 3A).The albumin secretion in urine was reduced by supplementing eucalyptol to diabetic mice (Figure 3B). Plasma insulin levels in eucalyptol-challenged db/db mice were lower than those in untreated db/db mice (Figure 3C).

**Figure 4:**
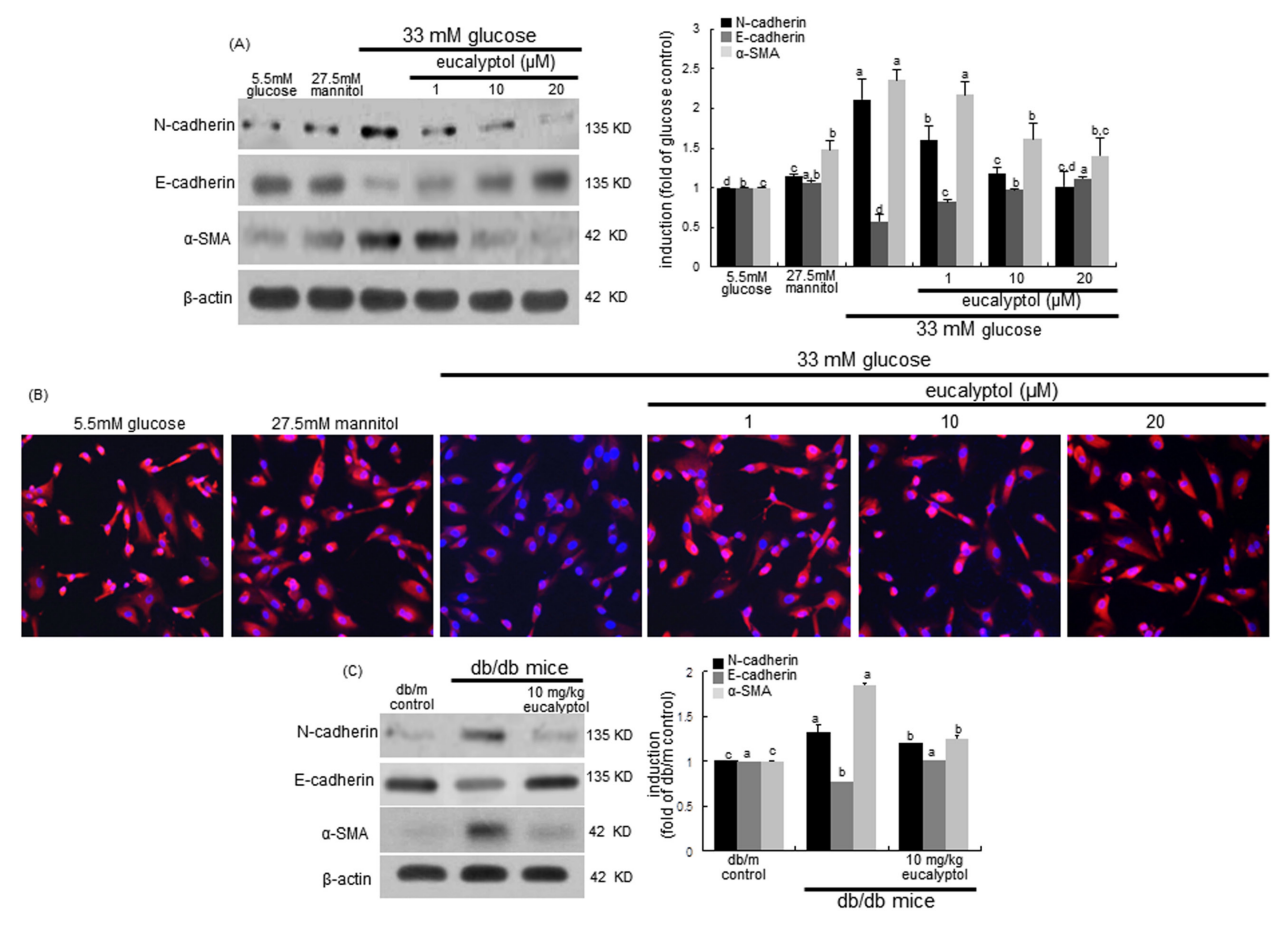
Western blot analysis (**A** and **C**) and immunocytochemial staining (**B**) showing inhibition of N-cadherin and α-SMA, and enhancement of E-cadherin by eucalyptol. Human renal proximal tubular epithelial cells were treated with 1-20 μM eucalyptol up to 72 h in the culture media of 33 mM glucose (A and B). Cells were also incubated in 5.5 mM glucose and 27.5 mM mannitol as osmotic controls. The db/db mice were supplemented with 10 mg/kg eucalyptol for 8 weeks (C). Cell lysates and renal cortical tissue extracts were subject to 8-12% SDS-PAGE and Western blot analysis with a primary antibody against N-cadherin, E-cadherin or α-SMA (A and C). Representative blot data were obtained, and β-actin protein was used as an internal control. The bar graphs (mean ± SEM, n = 3) in the right panels represent quantitative results of blots. Values not sharing a common letter are significantly different at P<0.05. Immunofluorescent analysis was performed with cy3 staining to examine E-cadherin induction by eucalyptol in high glucose-exposed cells (B). Nuclear counter-staining was done with 4’,6-diamidino-2-phenylindole. Magnification: 200-fold.

**Figure 5:**
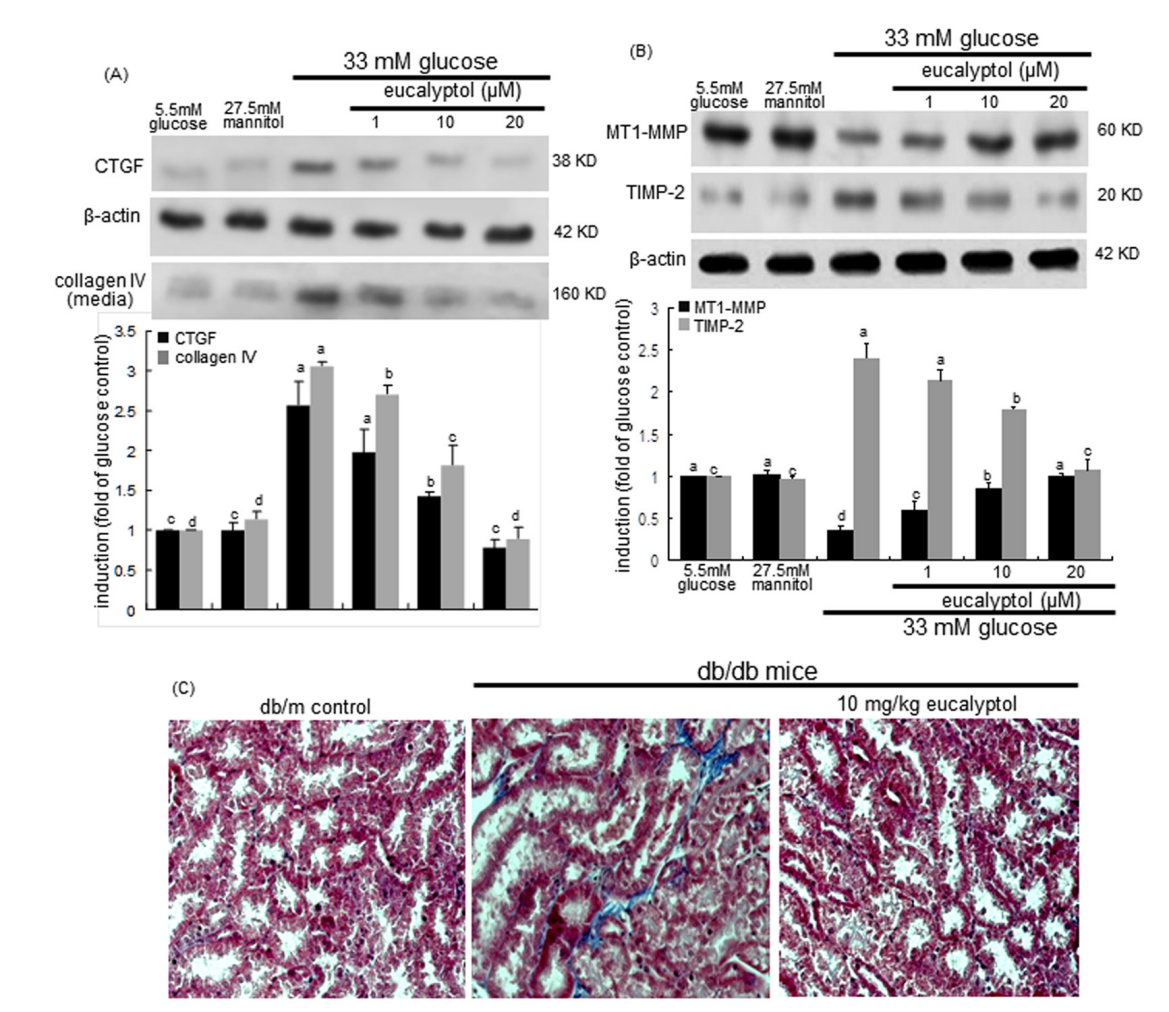
Effects of eucalyptol on CTGF induction and collagen IV secretion (**A**), MT1-MMP inhibition and TIMP-2 induction (**B**), and tubular formation of collagen fibers (**C**)**.** Human renal proximal tubular epithelial cells were treated with 1-20 μM eucalyptol in the culture media of 33 mM glucose for 72 h. Cells were also incubated in 5.5 mM glucose and 27.5 mM mannitol as osmotic controls. Western blot analysis with cell lysates or culture media was conducted with a primary antibody against CTGF, collagen IV, MT1-MMP or TIMP-2 (A and B). β-Actin protein was used as an internal control. The bar graphs (mean ± SEM, n=3) in the bottom panels represent quantitative desitometric results. Values not sharing a common letter are significantly different at P<0.05. The db/db mice were supplemented with 10 mg/kg eucalyptol for 8 weeks. Deposition of collagen fibers in db/m control and db/db mice was observed by Masson trichrome staining (C). The reddish staining indicates cytoplasm and muscle fibers and the blue staining shows collagen fibers and connective tissues. Each photograph is representative of four mice. Magnification: 400-fold.

**Figure 6:**
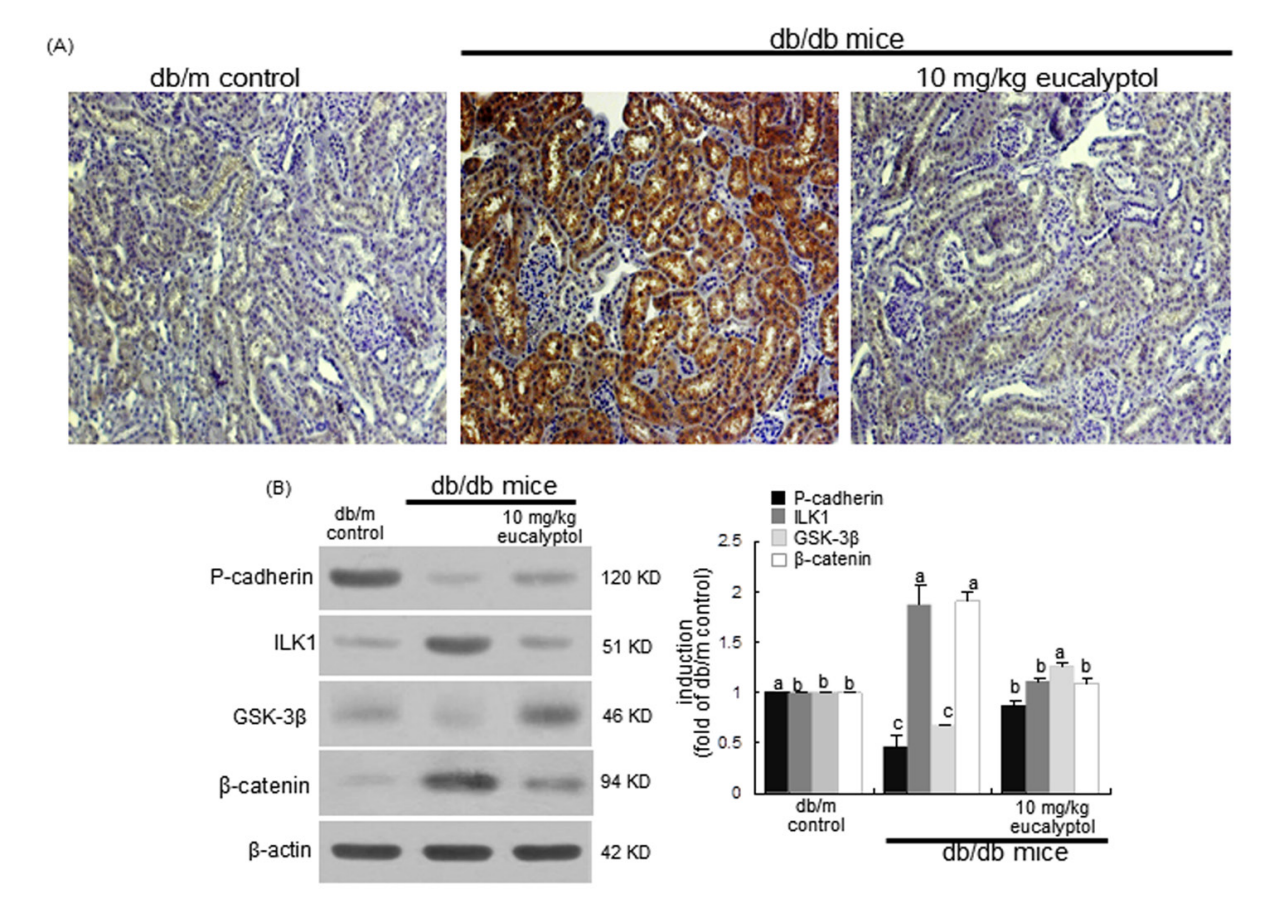
Modulation of proximal tubular induction of Snail1, P-cadherin, ILK1, GSK-3β and β-catenin by oral treatment of eucalyptol The db/db mice were orally administrated with 10 mg/kg eucalyptol for 8 weeks. Immunohistochemical staining was performed to observe proximal tubular Snail1 in db/db mice (**A**). Magnification: 200-fold. Tissue extracts were subject to 8-12% SDS-PAGE and Western blot analysis with a primary antibody against P-cadherin, ILK1, GSK-3β or β-catenin (**B**). β-Actin was used as internal control. The bar graphs (mean ± SEM, n=3) in the right panels represent quantitative desitometric results. Values not sharing a common letter are significantly different at P<0.05.

**Figure 7:**
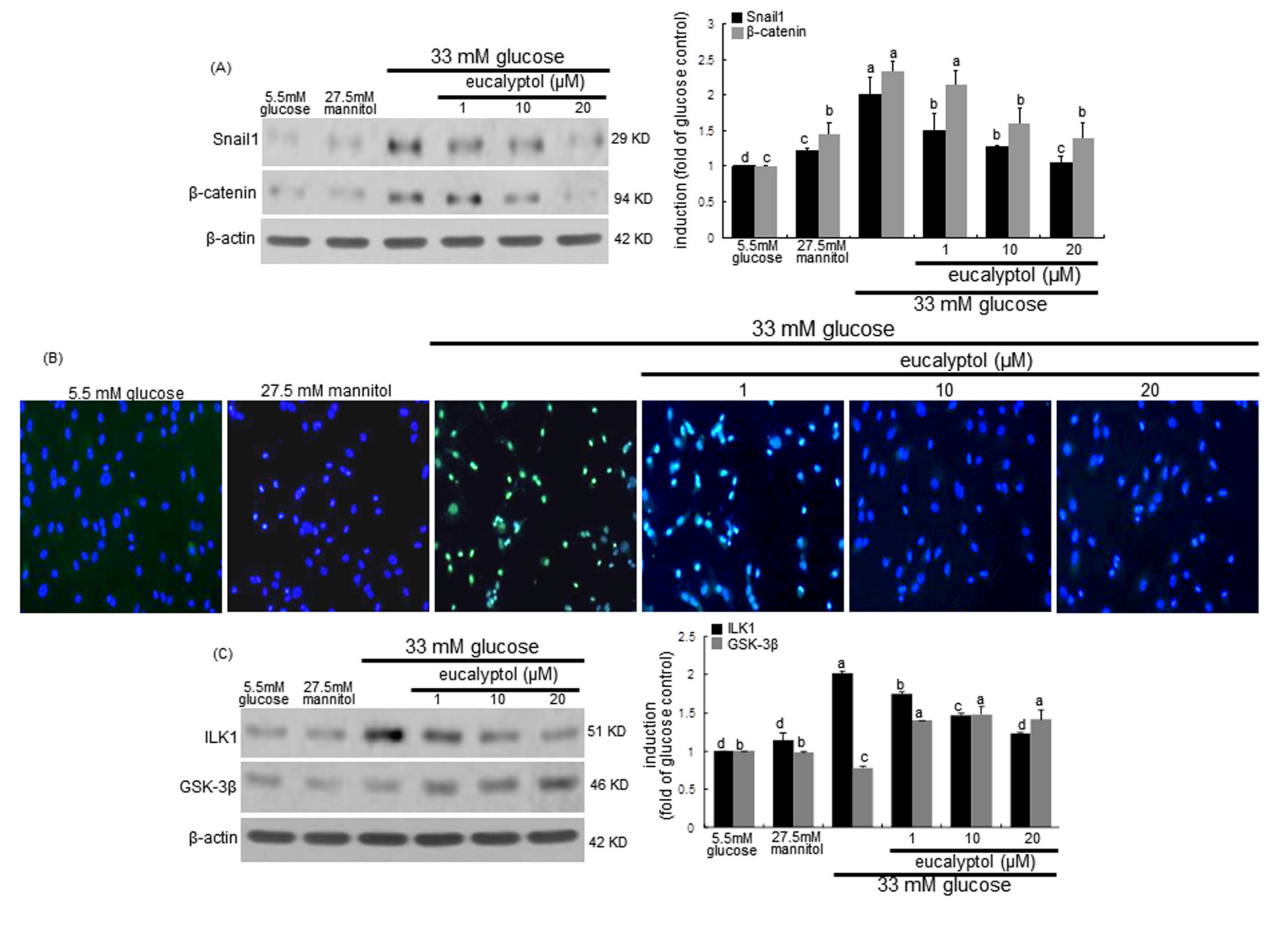
Western blot analysis and immunocytochemical staining showing modulation of the expression of Snail1, β-catenin, ILK1 and GSK-3β Human renal proximal tubular epithelial cells (RPTEC) were treated with 1-20 μM eucalyptol up to 72 h in the culture media of 33 mM glucose. Cells were also incubated in 5.5 mM glucose and 27.5 mM mannitol as osmotic controls. The db/db mice were supplemented with 10 mg/kg eucalyptol for 8 weeks. Cell lysates and renal cortical tissue extracts were subject to 8-12% SDS-PAGE and Western blot analysis with a primary antibody against Snail1, β-catenin, ILK1, or GSK-3β (**A** and **C**). β-Actin protein was used as an internal control. The bar graphs (mean ± SEM, n=3) in the right panels represent quantitative desitometric results. Values not sharing a common letter are significantly different at P<0.05. FITC-immunofluorescence analysis was performed to examine proximal tubular Snail1 induction in eucalyptol-treated RPTEC exposed to high glucose (**B**). Counter-staining was done with 4’,6-diamidino-2-phenylindole. Magnification: 200-fold.

**Figure 8:**
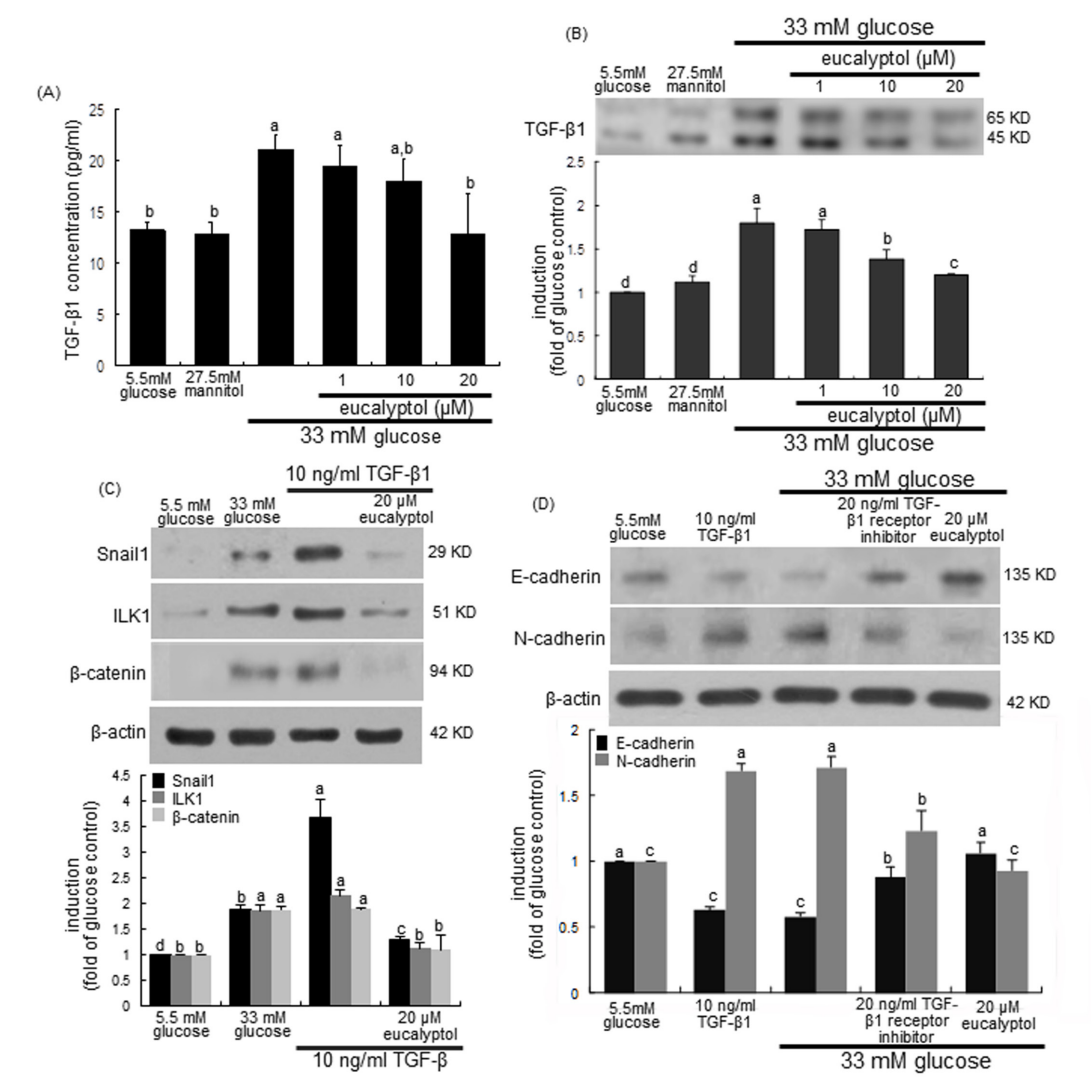
Effects of eucalyptol on TGF-β1 induction by high glucose (A and B), inhibition of TGF-β1-induced expression of Snail1, ILK1 and β-catenin by eucalyptol (C), and effects of TGF-β1 receptor inhibitor on expression of E-cadherin and N-cadherin (D) Human renal proximal tubular epithelial cells were treated with 1-20 μM eucalyptol for 72 h in the culture media of 33 mM glucose (A and B). Cells were also incubated in 5.5 mM glucose and 27.5 mM mannitol as osmotic controls. In other experiments, cells was cultured with 10 ng/ml TGF-β1 or 20 ng/ml TGF-β1 receptor inhibitor in the absence and presence of 20 μM eucalyptol for 72 h (C and D). TGF-β1 in culture media was measured by using an ELISA kit (A). Cell lysates were subject to 8-12% SDS-PAGE and Western blot analysis with a primary antibody against TGF-β1, Snail1, β-catenin, E-cadherin or N-cadherin (B, C and D). β-Actin protein was used as an internal control. The bar graphs (mean ± SEM, n=3) in the bottom panels represent quantitative desitometric results. Values not sharing a common letter are significantly different at P<0.05.

**Figure 9:**
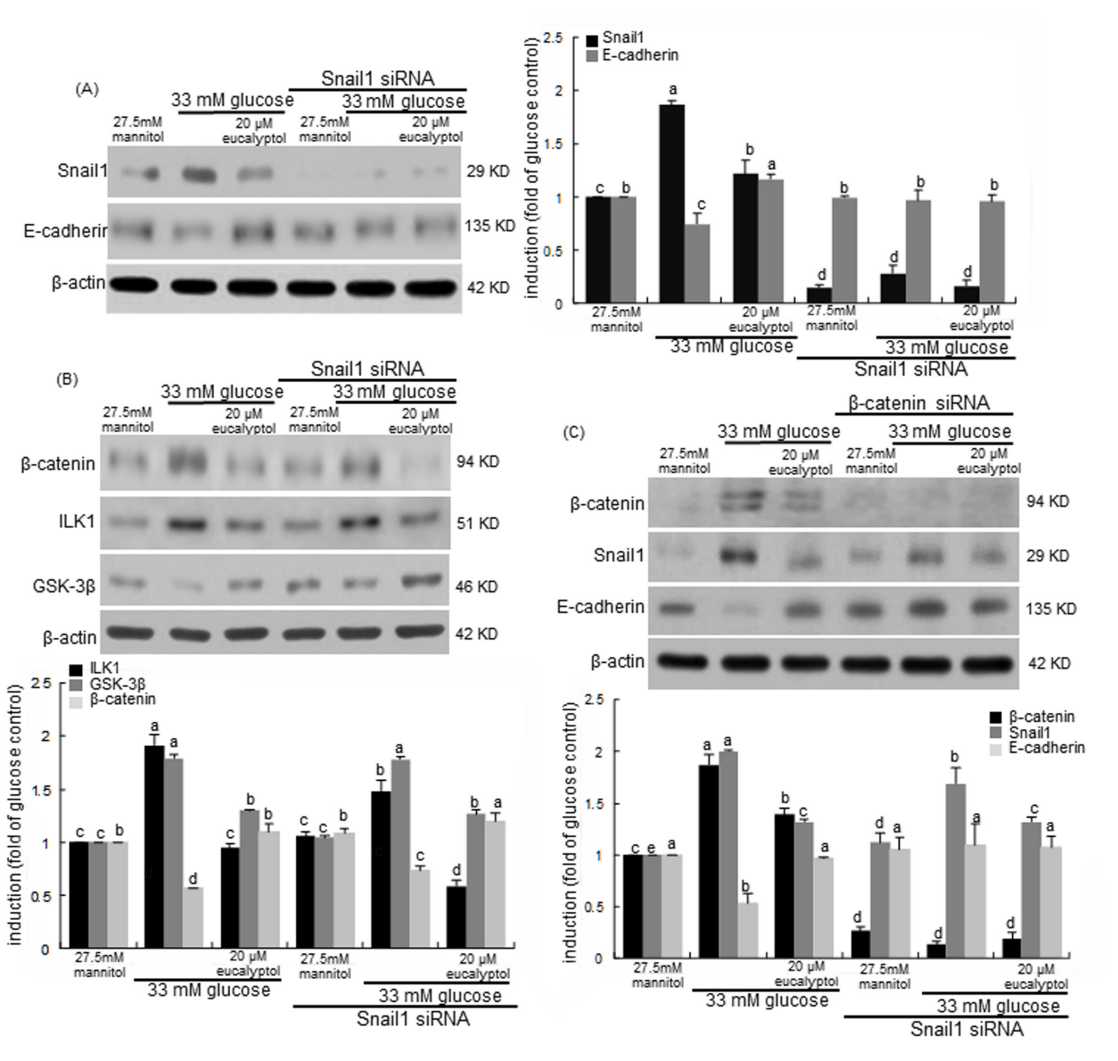
Effects of eucalyptol and silencing of Snail1 (A and B) and β-catenin (C) genes on expression of E-cadherin, GSK-3β, β-catenin and Snail1 in the presence of 33 mM glucose Snail1 siRNA- or β-catenin siRNA-transfected human renal proximal tubular epithelial cells were treated with 20 μM eucalyptol for 72 h in the culture media of 33 mM glucose. Transfected cells were also incubated in 27.5 mM mannitol as an osmotic control. Cell lysates were subject to 8-12% SDS-PAGE and Western blot analysis with a primary antibody against Snail1, E-cadherin, ILK-1, GSK-3β or β-catenin. β-Actin protein was used as an internal control. The bar graphs (mean ± SEM, n=3) in the right panel represent quantitative desitometric results. Values not sharing a common letter are significantly different at P<0.05.

### Western blot analysis

Western blot analysis was conducted using whole cell lysates and culture media prepared from RPTEC at a density of 3.5 x 10^5^ cells/dish, and renal cortical tissue extracts. Whole cell lysates and tissue extracts was prepared in a lysis buffer containing 1 M β-glycerophosphate, 1% β-mercaptoethanol, 0.5 M NaF, 0.1 M Na_3_VO_4_ and protease inhibitor cocktail. Cell lysates and tissue extracts containing equal amounts of proteins and equal volumes of culture media were electrophoresed on 8-12% SDS-PAGE and transferred onto a nitrocellulose membrane. Nonspecific binding was blocked with either 3% fatty acid-free bovine serum albumin or 5% nonfat dry skim milk for 3 h. The membrane was incubated overnight at 4°C with each primary antibody of target proteins and washed in a TBS-T buffer for 10 min. The membrane was then incubated for 1 h with a secondary antibody of goat anti-rabbit IgG, goat anti-mouse IgG, or donkey anti-goat IgG conjugated to HRP. Each target protein level was determined by using immobilon western chemiluminescent horseradish peroxidase substrate (Millipore Corp., Billerica, MA) and Agfa X-ray film (Agfa-Gevaert, Belgium). Incubation with monoclonal mouse β-actin antibody was also performed for comparative controls.

### Immunostaining

RPTEC (1.5 x 10^4^ cells) grown on 8-well glass chamber slides were exposed to 33 mM glucose in the absence and presence of 1-20 μM eucalyptol. RPTEC were fixed with 4% formaldehyde for 10 min and permeated with 0.1% Triton X-100 for 5 min on ice. Cells were blocked using a 4% FBS for 1 h. Immunofluorescent cytochemical staining of RPTEC was performed using E-cadherin antibody and Cy3-conjugated anti-rabbit IgG. In addition, immunofluorescent cytochemical staining for Snail1 was done with FITC -conjugated anti-rabbit lgG. Nuclear staining was performed with 4’,6-diamidino-2-phenylindole (DAPI). Each slide was mounted in VectaMount mounting medium (Vector Laboratories, Burlingame, CA). Images were taken using an optical Axiomager microscope system (Zeiss, Oberkochen, Germany). The protein levels of E-cadherin and Snail were quantified with an image analysis program from the microscope system.

For the immunohistochemical staining, kidney was obtained at the end of the experiments and fixed in 10% buffered formalin. The paraffin-embedded kidney tissues were sectioned at 5 μm thickness, deparaffinized, and dehydrated with xylene and graded ethanol solutions. Tissue sections were pre-incubated with 3% H_2_O_2_ for 5 min. Kidney tissue sections was incubated with a primary antibody of Snail1 overnight, treated with a secondary antibody conjugated to 3,3’-diaminobenzidine and counter-stained with hematoxylin for the nuclear staining. The stained tissue sections were mounted on slides and images were taken with an Axiomager Optical fluorescence microscope.

### Masson trichrome staining

For the histological analysis, kidney was obtained at the end of the experiments and fixed in 10% buffered formalin. The paraffin-embedded kidney tissues were sectioned at 5 μm thickness, de-paraffinized and stained with Masson trichrome for the light microscopic visualization of collagen fibers and muscle fibers. The stained tissue sections were examined using an optical Axiomager microscope, and three images were taken for each section.

### TGF-β1 production

The TGF-β1 secretion from RPTEC was determined by using enzyme-linked immunosorbent assay (ELISA). TGF-β1 secretion was measured in collected culture media by using a commercial ELISA kit (R&D System).

### Small interfering RNA (siRNA) transfection of Snail1 and β-catenin

To silence Snail1 and β-catenin genes in RPTEC, a stable transfection assay was conducted with 3 µg Snail1 siRNA and β-catenin siRNA (Santa Cruz Biotechnology) injected into the cells by using a commercial lipofectamine 3000 mixture (Life Technologies, Grand Island, NY). The Snail1 siRNA- or β-catenin siRNA-lipofectamine 3000 mixture was incubated with RPTEC for 24 h at 37°C. After the transfection, cells were treated with high glucose for 72 h in the absence and presence of 20 μM eucalyptol. Cells were lysed in a lysis buffer and Western blot analysis was conducted with anti-human Snail1, anti-human E-cadherin, anti-human GSK-3β and anti-human β-catenin.

### Statistical analysis

The data are presented as mean ± SEM for each treatment group. Statistical analyses were conducted using a Statistical Analysis Systems program (SAS Institute, Cary, NC). One-way ANOVA was used to determine inhibitory effects of eucalyptol on renal fibrosis in tubular epithelial cells and in diabetic mice. Differences among treatment groups were analyzed with Duncan’s multiple-range test and were considered to be significant at P<0.05.

## Abbreviations

CTGF: connective tissue growth factor
DN: diabetic nephropathy
ECM: extracellular matrix
EMT: epithelial to mesenchymal transition
GSK-3β: glycogen synthase kinase-3β
HbA1c: glycated hemoglobin
ILK1: integrin-linked kinase 1
MT1-MMP: membrane type 1-matrix metalloproteinase
RPTEC: human renal proximal tubular epithelial cells
α-SMA: α-smooth muscle actin
TGF-β1: transforming growth factor-β1
TIMP-2: tissue inhibitor of metalloproteinase-2
